# A standardized method to quantitatively analyze optical coherence tomography angiography images of the macular and peripapillary vessels

**DOI:** 10.1186/s40942-022-00426-9

**Published:** 2022-10-15

**Authors:** Luiz Guilherme Marchesi Mello, Taurino dos Santos Rodrigues Neto, Epitácio Dias da Silva Neto, Rony Carlos Preti, Mário Luiz Ribeiro Monteiro, Leandro Cabral Zacharias

**Affiliations:** 1 Department of Specialized Medicine, Centro de Ciências da Saúde (CCS), Universidade Federal do Espírito Santo, Vitória, Brazil; 2 Division of Ophthalmology and the Laboratory for Investigation in Ophthalmology (LIM-33), Faculdade de Medicina FMUSP, Universidade de São Paulo, Av. Dr Enéas de Carvalho Aguiar, 255, Cerqueira César, São Paulo, 05403-001 Brazil

**Keywords:** Angiography, Blood vessels, Optic disk, Optical coherence tomography, Retina

## Abstract

**Background:**

Optical coherence tomography angiography (OCTA) is a relatively new non-invasive imaging technique to evaluate retinal vascular complexes. However, there is still a lack of standardization and reproducibility of its quantitative evaluation. Furthermore, manual analysis of a large amount of OCTA images makes the process laborious, with greater data variability, and risk of bias. Therefore, the aim of this study is to describe a fast and reproducible quantitative analysis of the foveal avascular zone (FAZ), macular superficial and deep vascular complexes (mSVC and mDVC, respectively), and peripapillary superficial vascular complex (pSVC) in OCTA images.

**Methods:**

We survey models and methods used for studying retinal microvasculature, and software packages used to quantify microvascular networks. These programs have provided researchers with invaluable tools, but we estimate that they have collectively achieved low adoption rates, possibly due to complexity for unfamiliar researchers and nonstandard sets of quantification metrics. To address these existing limitations, we discuss opportunities to improve effectiveness, affordability, and reproducibility of microvascular network quantification with the development of an automated method to analyze the vessels and better serve the current and future needs of microvascular research. OCTA images of the macula (10°x10°, 15°x15°, or 20°x20° centered on the fovea) and peripapillary area (15 × 15º centered on optic nerve head) were exported from the device and processed using the open-source software Fiji. The mSVC, mDVC, and pSVC were automatically analyzed regarding vascular density in the total area and four sectors (superior, inferior, nasal, and temporal). We also analyzed the FAZ regarding its area, perimeter, and circularity in the SVC and DVC images.

**Results:**

We developed an automated model and discussed a step by step method to analyze vessel density and FAZ of the macular SVC and DVC, acquired with OCTA using different fields of view. We also developed an automated analysis of the peripapillary SVC.

**Conclusion:**

Our developed automated analysis of macular and peripapillary OCTA images will allow a fast, reproducible, and precise quantification of SVC, DVC, and FAZ. It would also allow more accurate comparisons between different studies and streamlines the processing of images from multiple patients with a single command.

## Introduction

Optical coherence tomography angiography (OCTA) has emerged in the last years as a useful noninvasive imaging modality to assess ocular vasculature. Briefly, OCTA consists of repeated acquisitions of optical coherence tomography (OCT) scans of the same spot on the retina and choroid to evaluate temporal changes in the backscattered signal caused by moving blood particles moving within the vessels. Using an image processing algorithm, an en face image of the vascular network of the analyzed region is created and, if desirable, segmented in different vascular complexes [1].

Since the advent of OCTA, the risk of acquisition errors has been reduced by using eye-tracking systems, faster acquisition software, and higher resolution. However, the procedure is still hampered by the long time required for the repetitive scans (especially when larger areas are analyzed), the impossibility of detecting vascular leakage or permeability abnormalities, differences between software, and the persistence of image artifacts [1]. Originally, the interpretation of OCTA images was essentially qualitative, but the introduction of novel quantitative vascular network parameters has contributed to diagnosis, prognosis, and monitoring of patients [2, 3].

ImageJ (National Institutes of Health, Bethesda, MD, USA) [4] is an open-source software commonly used in manual or automatic image analyses, including OCTA [5]. OCTA images can be processed in different manners using ImageJ to obtain quantitative data, [6, 7] but the current lack of detailed information makes it difficult to establish a standard method of reproducing macular and peripapillary findings [6–9]. The purpose of this paper is to review the literature on quantitative OCTA analysis and propose a standard method of comprehensive quantitative processing and analysis of macular and peripapillary OCTA images, increasing the comparability of studies using compatible OCTA equipment.

## Methods

### Study participants

This cross-sectional, observational, and descriptive study complied with the Declaration of Helsinki (1996), the Nuremberg Code (1947), the guidelines of the National Health Council on research involving humans (Resolution 466/12), and our Institutional Review Board Ethics Committee. All subjects gave their informed written consent before enrolling in the study.

The inclusion criteria required each subject to be a consenting adult (≥ 18 years) and have healthy eyes. The exclusion criteria were: diabetes mellitus, serious chronic systemic disease, previous brain surgery, ocular surgery, ocular diseases capable of affecting the retina, choroid or optic nerve (retinopathies, uveitis, optic neuropathies or abnormalities), high myopia (axial length > 26.5 or spherical refraction <-6 diopters), high hypermetropia (spherical refraction > + 6 diopters) and cylinder refraction > ± 3 diopters, intraocular pressure > 21 mmHg, media opacity compromising the quality OCT/OCTA scans (corneal opacities, nuclear opalescence > 2 according to the Lens Opacities Classification System III, and vitreous opacities), and best-corrected visual acuity (VA) worse than 20/30.

### Ophthalmological examination and OCTA image acquisition

All patients underwent a complete ophthalmological examination, including VA evaluation, slit-lamp biomicroscopy, Goldman applanation tonometry, fundoscopy, OCT and OCTA, and ocular biometry for axial length measurement, if necessary (IOL Master 500; Carl Zeiss Meditec, Germany). After the VA measurements, the pupils were dilated with 1% tropicamide eye drops to perform a complete fundus examination and to acquire high-quality OCT scans. Spectral-domain OCT scans were acquired using the Spectralis® OCT module (Heidelberg Engineering, GmbH, Heidelberg, Germany). Macular and peripapillary scans were acquired using predetermined automatic real-time tracking (ART) for each type of acquisition, with a quality index of at least 25. Images with many artifacts due to movement, projection, duplicated vessels, or distortions were repeated and all scans were manually reviewed to ensure adequate segmentation.

The macular protocol consisted of a 10º x 10º, 15º x 15º, or 20º x 20º OCTA scan (512 A-scans/B-scan and 512 B-scans/volume) centered on the fovea. The foveal center was manually determined and confirmed by checking the OCT B-scans acquired with the OCTA scanning protocol. The optic nerve head protocol consisted of a 15º x 15º OCTA scan (512 A-scans/B-scan and 512 B-scans/ volume) centered on the optic disc. En face OCTA images of the superficial vascular complex (SVC) and deep vascular complex (DVC) were generated using the automatic retinal layer segmentation of the Spectralis® software. The upper and lower limits of the SVC were the internal limiting membrane and a point 17 μm above the lower edge of the inner plexiform layer, respectively. Similarly, the upper and lower limits of the DVC were a point 17 μm above the lower edge of the inner plexiform layer and the extremity of the outer plexiform layer, respectively.

### Qualitative protocol

Two masked examiners reviewed all images independently. Scans were excluded in the presence of any of the following: (i) insufficient resolution, (ii) weak local signal caused by artifacts such as visual floaters, (iii) residual motion artifacts visible as irregular vessel patterns or disc boundaries in the en face angiogram, and (iv) off-centered fovea. Discrepancies between the two reviewers were resolved by consensus or adjudication by an experienced third reviewer.

We also reviewed the literature for a brief discussion on OCTA imaging processes, including the physical principles and algorithms of Heidelberg OCTA. Pubmed and Google Scholar were searched for quantitative microvascular OCTA analyses, automated and manual thresholding algorithms for macular and peripapillary OCTA, objective OCTA-based evaluations of the size and shape of the foveal avascular zone (FAZ) in normal subjects, and OCTA studies employing the ‘Level Sets’ macro (a plug-in used to progressively evaluate differences between adjacent pixels; available at https://imagej.net/Level_Sets).

Most available software segregates the retinal vasculature into a ‘superficial slab’ and a ‘deep slab’ slab. Several field-of-view options may be provided, but as the field of view increases, the resolution of the scan decreases since the same number of A-scans are being used to scan a larger area. The predefined retinal slabs shown in the output images may be analyzed quantitatively with external software. Unfortunately, measuring and quality control methods have not been standardized, making it difficult to compare studies, and metric calculations vary from study to study. Recent studies have compared different methods of assessing capillary density and morphology [6, 7]. OCTA scans acquired for quantitative analysis are highly dependent on image processing. Pioneering studies proposed the best thresholds to intra-class correlation coefficients between consecutive OCTA measurements. Our group has proposed a Heidelberg OCTA-based automated quantification method for the assessment of retinal vasculature in healthy subjects, standardizing and streamlining analysis by processing images from multiple patients with a single command. The method of quantitative analysis is described step by step below, followed by a discussion on the methods employed in previous studies.

### OCTA image processing

**Fig. 1:**
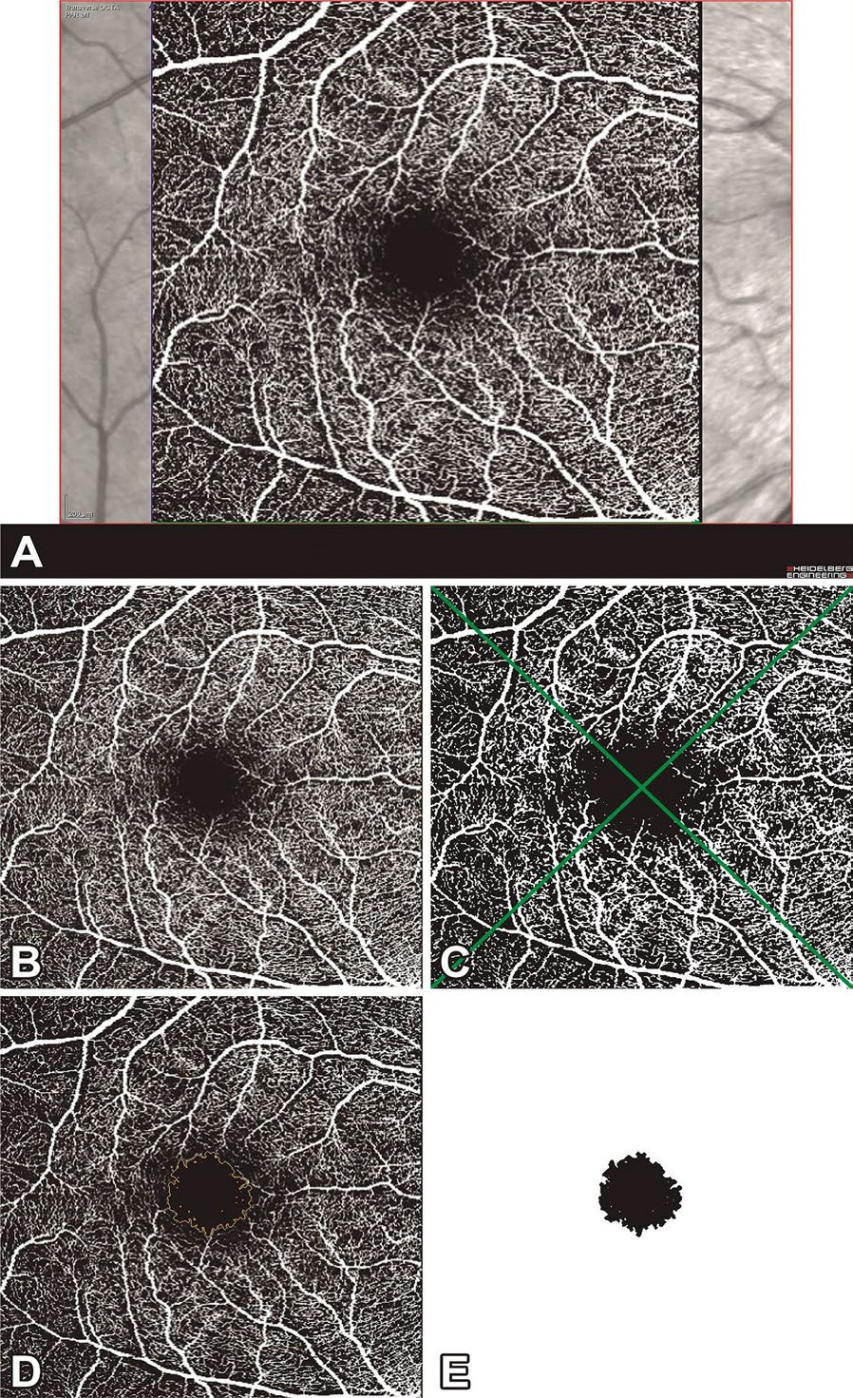
Optical coherence tomography angiography scan of the macular superficial vascular complex of the right eye (**A**) and its post-process images (**B**–**E**). The OCTA is cropped to keep only the retinal vascular complex (**B**) and then is modified after Otsu thresholding operation (**C**), dividing into four sectors (superior, inferior, nasal, and temporal) for the analysis. The foveal avascular zone is automatically delimited (**D**) from the cropped image (**B**) using the Level Sets macro, generating an image containing the foveal avascular zone after removing the area beyond it (**E**)

En face OCTA images were used to calculate the FAZ and the vascular density of all complexes (macular SVC and DVC, and peripapillary SVC) using ImageJ (National Institutes of Health, Bethesda, Maryland, USA; available at https://imagej.nih.gov/ij/download.html). OCTA reports of macular SVC (mSVC) and macular DVC (mDVC) were exported in TIFF format, centered on the fovea, and cropped to 962 × 962 pixels (Fig. 1A, B). Subsequently, the images were binarized to black and white and Otsu’s thresholding method 10 was used for vascular density analysis (Fig. 1 C). The white pixels were considered vessels and the overall density was calculated by dividing the vessel area by the total area of interest, expressed according to the sector (superior, inferior, nasal, and temporal). We developed a macro (sequence of commands and functions stored in a file module, serving as a shortcut) for the analysis of mSVC and mDVC in multiple OCTA images captured using different fields of view (macro files are available in a public repository—see Data availability statement). The FAZ of the mSVC and mDVC were delimited automatically (Fig. 1D) and segmented (Fig. 1E) from the cropped macular OCTA image (Fig. 1B) using the Level Sets macro, which automatically measures and outputs the FAZ metrics (area, perimeter, and circularity).

**Fig. 2:**
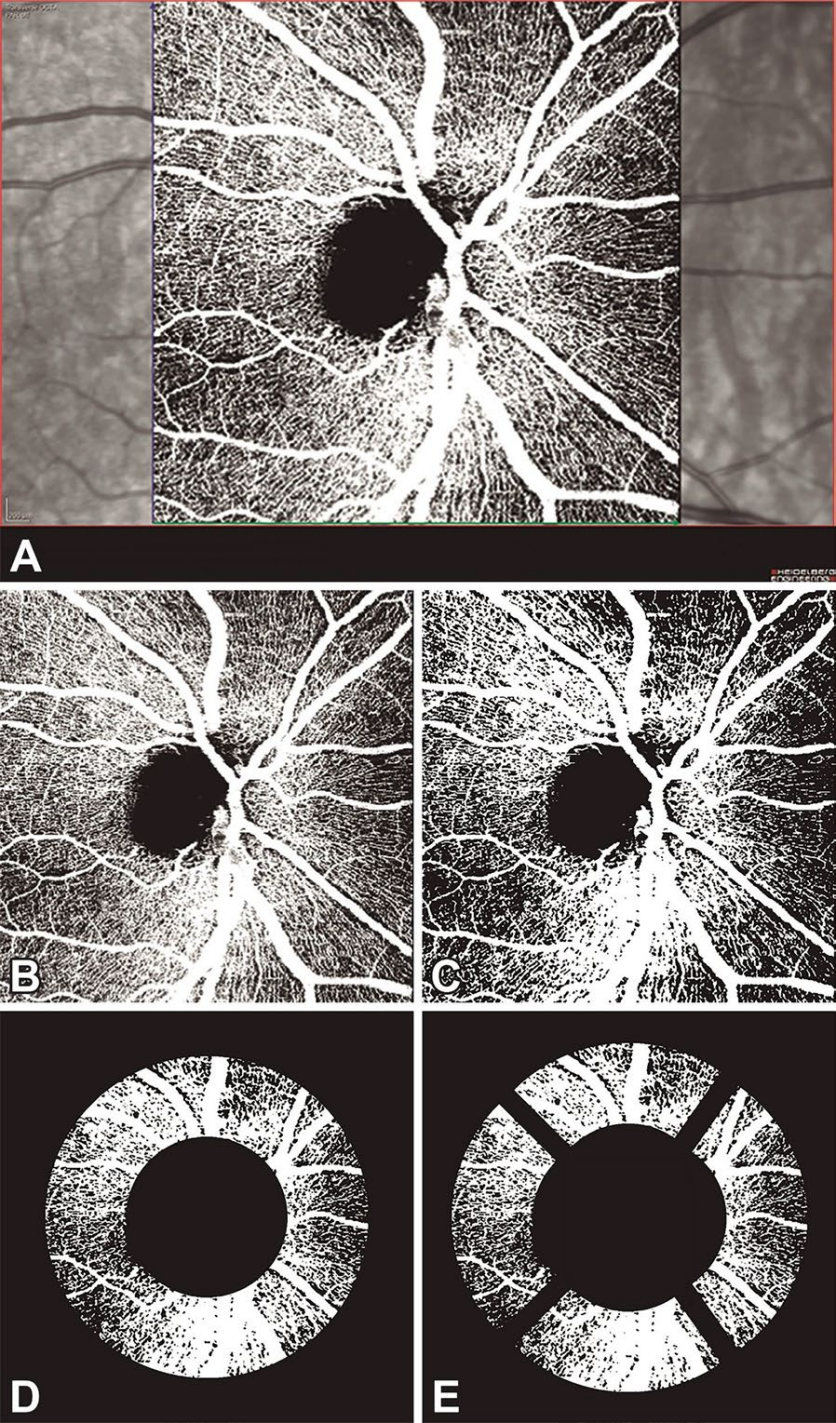
Optical coherence tomography angiography scan of the peripapillary superficial vascular complex of the right eye (**A**) and its post-process images (**B**–**E**). The OCTA is cropped to keep only the retinal vascular complex (**B**) and then is modified after Otsu thresholding operation (**C**), dividing into a ring (**D**) and four sectors (superior, inferior, nasal, and temporal) for the analysis. Panel E shows the four sectors of the peripapillary ring slightly separated from each other

We created another macro to analyze FAZ of the SVC and DVC of multiple OCTA images in different fields of view (macro files are available in a public repository—see Data availability statement). OCTA reports of peripapillary SVC (pSVC) were exported from OCT in TIFF format, centered on the optic nerve head, and cropped to 938 × 938 pixels (Fig. 2A, B). As with macular OCTA images, the cropped peripapillary OCTA images were binarized with Otsu’s thresholding algorithm for vascular density analysis (Fig. 2C). The pSVC analysis was based on an annular area of 1.7 mm internal diameter and 3.4 mm external diameter centered on the optic nerve head (Fig. 2D). The pSVC ring was analyzed concerning total average (360º) and sectors (inferior 80°, superior 80°, nasal 110°, temporal 90°), corresponding to the sectors of the peripapillary retinal nerve fiber layers. We also created an automated macro to analyze the 15° x 15° pSVC. The macro files are available for download (macro files are available in a public repository—see Data availability statement).

### Data analysis and statistics

The descriptive statistics included mean ± standard deviation (SD) for normally distributed variables. The Shapiro-Wilk test was used to assess normality.

## Results

Thirty-seven healthy participants were enrolled in this study, aged 44 years on average. The mean axial length was 23.19 mm (right eye) and 23.05 mm (left eye), and the mean intraocular pressure was 15 mmHg. The mean signal quality (Q) was 41 for 10º x 10º, 39 for 20º x 20º, and 35 for peripapillary 15° x 15°. The procedure used to analyze the FAZs of multiple images comprises the following steps:

### Step-by-step FAZ analysis

First step: Save OCTA images in tiff format on a computer, segregating OCTAs into deep and superficial FAZ, and assign a name to the file containing code, eye (right eye/left eye—OD/OS), FAZ, and layer (deep/superficial). Ex: “01_OD_FAZ_SUPERFICIAL”.

Second step: Install ImageJ, then add the “Read and Write Excel” plugin to ImageJ (see https://imagej.net/Read_and_Write_Excel; all plugin files are also available in a public repository—see Data availability statement). This plugin allows exporting the results to Excel.

Third step: Using the image editor (ImageJ), crop the OCTA area to the size of the “15 × 15_cropped.tif” file (962 × 962 pixels).

**Fig. 3:**
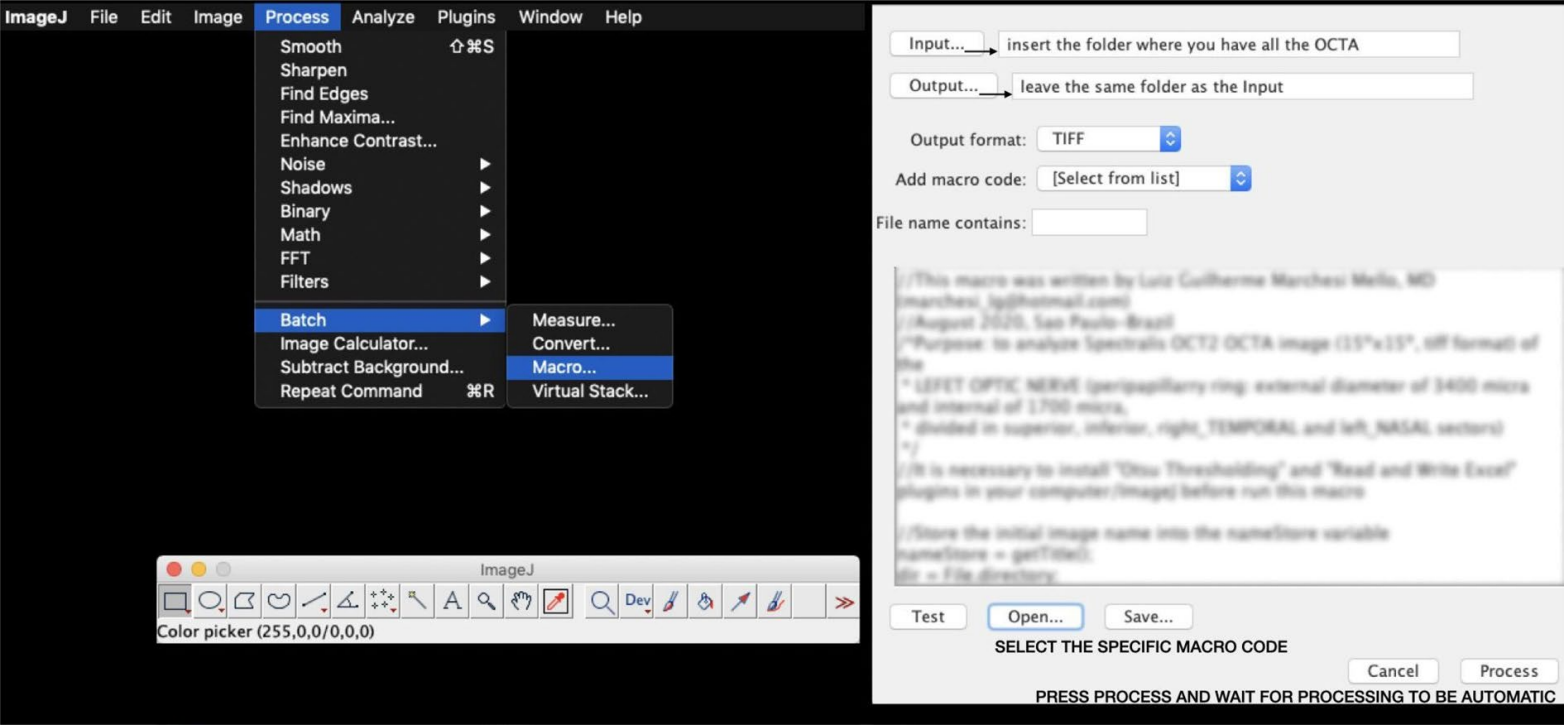
Image J commands to select the macros and process the images

Fourth step: Execute the ImageJ commands: Process > Batch > Macro > Input (folder containing the OCTAs of FAZs to be evaluated) / Output (save images generated by ImageJ in the same folder as Input) / Select output format: tiff > Click in the “Open…” button and select file with macro code for a specific FAZ of 10 × 10 or 15 × 15 or 20 × 20 from SVC or DVC (e.g., “FAZ_10 × 10_DVC.ijm” or “FAZ_10 × 10_SVC.ijm”) > Start automatic processing (click in the “Process” button) > View result expressed in area (mm2), perimeter (mm) and circularity on Excel spreadsheet on the desktop (Fig. 3).

Fifth step: Rename the excel file and remove it from the desktop. ‘Area’ is the size of the segmented FAZ, while ‘perimeter’ is the length of the FAZ contour. The circularity index (deviation relative to a perfect circle) is calculated with the formula: 4π(area/perimeter2). The closer to 0, the less circular and more irregular the shape. Post-processed images (boundaries and FAZ drawing) are automatically saved in the same folder as the original files (see Table 1).

**Table 1:** Normal values of foveal avascular zone of all complexes of 20 × 20 and 10 × 10 acquisition mode

	FAZ 10 × 10 (mm^2^)	FAZ 20 × 20 (mm^2^)	PERIMETER 10 × 10 (mm)	PERIMETER 20 × 20 (mm)	CIRC 10 × 10	CIRC 20 × 20
	Superficial deep	Superficial deep	Superficial deep	Superficial deep	Superficial deep	Superficial deep
Right eye	0.08 (± 0.002) 0.09 (± 0.002)	0.33 (± 0.03) 0.33 (± 0.01)	1.201 (± 0.00) 1.201 (± 0.00)	2.33 (± 0.09) 2.33 (± 0.00)	0.75 (± 0.01) 0.75 (± 0.02)	0.77 (± 0.01) 0.77 (± 0.00)
Left eye	0.09 (± 0.002) 0.09 (± 0.002)	0.33 (± 0.03) 0.33 (± 0.03)	1.201 (± 0.00) 1.201 (± 0.00)	2.33 (± 0.00) 2.33 (± 0.00)	0.74 (± 0.02) 0.75 (± 0.02)	0.77 (± 0.00) 0.78 (± 0.01)

The procedure used to analyze the vascular density of all complexes in multiple images comprises the following steps:

### Step-by-step macular vascular density analysis

First step: After saving the images as described in the previous section, segregate OCTAs according to the eye (“Macula OD” or “Macula OS”) and complexes (superficial/deep) for each eye, facilitating subsequent data processing (right/left sector inversion).

Second step: Assign a name to the OCTAs within each folder specifying patient code, eye (OD/OS), macula, and complexes (deep/superficial) (Ex: “01_OD_Macula_DEEP”). Using the image editor (ImageJ), crop the OCTA area to the size of the “15 × 15_cropped.tif” file (962 × 962 pixels).

Third step: Install the ImageJ plugins ‘Otsu_Thresholding.jar’ and ‘Read and Write Excel’ by putting the respective files in the folder “…\ImageJ.app\plugins” (plugin files are available in a public repository—see Data availability statement).

Fourth step: Go to Process > Batch > Macro > Input (place the folder together with the OCTA images to be evaluated) / Output (save images generated by ImageJ in the same folder as Input) > Select output format: tiff > Click in the “Open…” button and select file with macro code for a specific macula of 10 × 10 or 15 × 15 or 20 × 20 (Ex: “Macula_15 × 15.ijm”) > Click “Process” button (click ‘Ok’ each time the message “Found threshold:…” is displayed). View result expressed as density (% pixels) in each sector (total, top, bottom, left, right) on an Excel spreadsheet on the Desktop. Note that in OD, ‘left’ corresponds to ‘temporal’ and ‘right’ corresponds to ‘nasal’, while the opposite is true for OS. Rename the Excel file and remove it from the desktop. Post-processed threshold images are saved in the same folder as the original files (see Tables 2 and 3).

**Table 2:** Normal values of vascular density of 20 × 20 acquisition mode stratified by macular sectors

	Global (%)	Superior (%)	Inferior (%)	Nasal (%)	Temporal (%)
	Superficial deep	Superficial deep	Superficial deep	Superficial deep	Superficial deep
Right eye	24.08 (± 5.05) 22.26 (± 3.50)	24.25 (± 5.72) 20.05 (± 4.69)	33.88 (± 8.60) 20.48 (± 5.03)	33.88 (± 6.01) 22.73 (± 5.21)	15.55 (± 6.85) 25.21 (± 5.43)
Left eye	26.61 (± 4.87) 21.98 (± 5.17)	26.16 (± 5.84) 19.77 (± 4.87)	24.94 (± 5.48) 27.11 (± 5.76)	41.07 (± 9.61) 27.14 (± 6.17)	13.96 (± 4.58) 21.92 (± 6.04)

**Table 3:** Normal values of vascular density of 10 × 10 acquisition mode stratified by macular sectors

	Global (%)	Superior (%)	Inferior (%)	Nasal (%)	Temporal (%)
	Superficial deep	Superficial deep	Superficial deep	Superficial deep	Superficial deep
Right eye	24.41 (± 4.32) 23.87 (± 4.25)	25.86 (± 4.86) 23.44 (± 4.83)	26.38 (± 5.45) 25.68 (± 4.81)	27.16 (± 6.27) 25.68 (± 4.81)	20.89 (± 4.52) 25.09 (± 4.20)
Left eye	25.08 (± 3.82) 24.71 (± 3.50)	25.50 (± 4.79) 24.48 (± 4.41)	23.96 (± 4.92) 23.57 (± 4.70)	28.12 (± 4.67) 27.86 (± 3.94)	19.03 (± 4.01) 23.66 (± 4.07)

The procedure used to analyze the peripapillary vascular density of all complexes of multiple images comprises the following steps:

### Step-by-step peripapillary vascular density analysis

First step: After saving the images as described in the previous section, segregate OCTAs according to the eye (‘Nerve OD’ or ‘Nerve OS’), facilitating the subsequent data processing. Assign a name to the OCTAs within each folder specifying patient code, eye (OD/OS), and Nerve (Ex: “01_OD_Nerve”).

Second step: open ImageJ and go to Process > Batch > Macro > Input (folder containing all the OCTAs of the right eye nerves to be evaluated)/ Output (save images generated by ImageJ in the same folder as Input) > choose the output format: tiff > Click in the “Open…” button the file with the macro code for right eye nerve (Ex: “ONH_15 × 15_OD.ijm”). Press Process (click ‘Ok’ each time the message “Found threshold:…” is displayed). View the result expressed in density (% pixels) for the evaluated area (total ring, superior_rim, inferior_rim, left_temporal_OD_rim, right_nasal_OS_rim) on an Excel spreadsheet on the desktop.

Third step: Rename the Excel file and remove it from the desktop. The optic nerve division adopted is that of Suzuki et al.8 Density (% pixels) in the total area of ​​the peripapillary ring (360º), the upper rim (80º), the lower rim (80º), the left rim (temporal rim OD) (90º) / (nasal rim OS) (110º), right rim (nasal rim OD) (110º) / (temporal rim OS) (90º) (Table 4).

**Table 4:** Normal values of vascular density of 15 × 15 acquisition mode stratified by peripapillary sectors

	Global (%)	Superior (%)	Inferior (%)	Nasal (%)	Temporal (%)
	Superficial	Superficial	Superficial	Superficial	Superficial
Right eye	56.95 (± 8.62)	67.68 (± 14.13)	75.33 (± 12.49)	47.32 (± 12.04)	42.94 (± 17.24)
Left eye	55.29 (± 8.24)	68.73 (± 12.89)	74.90 (± 11.74)	49.64 (± 13.59	38.77 (± 11.82)

## Discussion

To our knowledge, this is the first study to standardize a method for quantitative analysis of Heidelberg OCTA Images of the macular and peripapillary vessels. Automated algorithms generate more reproducible results and thus allow for more accurate discrimination between healthy and pathological structures.

The quantitative analysis of macular and peripapillary OCTA images depends on image quality. Some authors believe noise and vessel discontinuities can impact on the quantitative parameters [10]. Indeed, OCTA scans with many image distortion should be excluded from the analysis [11, 12]. No minimum quality level has been established for the selection of OCTA images, but contrast quality, vessel continuity, and background noise level in the nonvascular area should be taken into account [13, 14]. In this study, we selected only high-quality images for analysis: after selecting images with satisfactory quality parameters, the mean signal quality (Q) was 41 for 10º x 10º, 39 for 20º x 20º, and 35 for peripapillary 15° x 15°. Thus, we suggest that exams with Q over such parameters can be considered as good quality in the Heidelberg OCTA. Furthermore, Q values obtained by the different OCTA models may differ, so each model should ideally describe the minimum image quality index for the best segmentation result.

Quantitative OCTA measurements are highly dependent on binarization and since higher levels of background noise can affect the binarization threshold, it is advisable to conduct background noise and continuous vessel segmentation analysis before binarization [13]. Image processing is an important step when calculating quantitative global vessel density from OCTA images. The choice of threshold to be used for image binarization is of utmost importance. Most researchers recommend manual and automated binarization methods using open-source software, such as ImageJ. Manual methods employ a binarization threshold based on the mean signal within the vessel-free FAZ. Automated algorithms use the histogram of the image to obtain a threshold. Rabiolo et al. found significant differences in vessel density between manual and automated binarization methods, compromising the reliability of consecutive examinations [15].

Differences in binarization thresholding methodologies have been shown to influence the quantification of OCTAs in healthy eyes. Automated should be preferred over manual binarization, but algorithms are not interchangeable and outcomes can differ significantly. In one study, the intra-class correlation coefficients between two consecutive OCTA measurements were significantly higher when the Otsu binarization method was used. The method minimizes variance between foreground and background structures in the image histogram [6]. In a previous qualitative analysis of binarization threshold methods in diabetic retinopathy patients, the overall Otsu threshold yielded the best results [16].

Data obtained with different settings are not easily compared. Interstudy comparability requires the adoption of a set of common methods and standards. Our group proposes a method of a quantitative assessment of retinal vasculature in healthy subjects using Heidelberg OCTA. The step-by-step algorithm was found to provide reliable quantitative vascular measurements of the macula and peripapillary networks. Moreover, the ImageJ macro script is low-cost, time-saving, accessible, and widely available. The considerable inter-method variability observed in quantitative OCTA outcomes in general highlights the difficulty in comparing studies and the potential of the macro presented in this paper. However, despite the number of steps involved, we believe the procedures are easy to learn and that the algorithm can be used to standardize quantitative OCTA measurements for greater reproducibility and comparability.

The FAZ is highly susceptible to retinal vascular changes. Accurate observations of area and regularity are necessary for an adequate understanding of normality, but few quantitative studies are available and measuring methods have not been standardized. The method proposed in this paper allows for objective and reproducible measurements. Manually establishing the boundary of the FAZ can be taxing and time-consuming, and manual segmentation tends to be closer to a circle and smoother than automated algorithms, resulting in a shorter perimeter and greater circularity [17].

The shape of the FAZ on OCTA is considered a good indicator of retinal pathology. Thus, in some recent studies, normal eyes and eyes with diabetic retinopathy were shown to differ significantly with regard to the circularity of the FAZ [18, 19]. The circularity of the FAZ can be expressed mathematically as a deviation from the perfect circle (1.0), using the equation: circularity = 4π(area/perimeter2). The lower the value, the greater the deviation. Since morphological FAZ parameters are less affected by individual variation than FAZ size in normal eyes, parameters related to the shape of the FAZ are likely better for monitoring the FAZ in patients with suspected disease [20]. Since the FAZ area depends on the OCT device used, measurements obtained with different devices should not be used in clinical trials [21, 22]. In the present study, we used Spectralis® OCTA to obtain the mean (± SD) FAZ area measurements shown in Table 1.

OCTA allows to non-invasively and quantitatively assess blood flow at the optic nerve head. Recent studies have shown that OCTA-measured retinal circumpapillary vessel density may be affected even in patients with non-primarily vascular optic nerve diseases such as glaucoma, inflammatory or hereditary optic neuropathies [23]. OCTA scanning makes it relatively easy to evaluate the peripapillary vascular network. A dense microvascular network with no focal capillary dropout can be observed around most healthy optic discs (often more visible immediately adjacent to the border of the disc), with decreasing clarity towards the periphery. By analyzing the vessel density separately in different sectors and layers of the optic nerve, OCTA helps distinguish between healthy and damaged optic nerves and potentially sheds light on the pathogenesis of optic disc diseases [17]. Based on our results, we propose a practical and automated method of obtaining quantitative perivascular vessel density data using Heidelberg OCTA and present normal ranges for each optic disc sector.

Our study was limited by the relatively small number of subjects and the use of only one measurement per eye. Vascular complexes were the focus of this paper, clinically it would be more interesting if we could segment and evaluate each plexus. Moreover, we did not assess comparability and reproducibility across different OCTA devices, and only normal eyes were included in the sample. It is possible that retinal layer changing in retinal pathologies may be a problem for adequate segmentation and algorithm performance. Although we have facilitated the quantitative analysis of the images, it is still necessary to export the image from the device and use third-party software. Ideally, the automated analysis strategy should be incorporated as an update in the device itself.

In conclusion, the standardization of OCTA measuring methods is of great importance for the scientific and clinical use of OCTA. The proposed steps of OCTA-based assessment of macular and peripapillary parameters (SVC, DVC, FAZ) were found to be reproducible, accurate, and easy to learn. The adoption of our method would favor inter-study comparability and contribute to the current understanding of a range of pathologies and treatment responses.
